# Post-traumatic Dupuytren’s contracture in a paediatric patient: a case report and literature review

**DOI:** 10.1080/23320885.2024.2436678

**Published:** 2024-12-11

**Authors:** Andia Soltani, Alexander Zargaran, Norbert Kang

**Affiliations:** a Department of Plastic and Reconstructive Surgery, Royal Free London NHS Foundation Trust, London, United Kingdom; b University College London, London, United Kingdom; c Aston University, Birmingham, United Kingdom

**Keywords:** Dupuytren’s disease, Dupuytren’s contracture, finger flexion deformity, paediatric hand conditions

## Abstract

Dupuytren’s disease is rare in children. We present the case of a 14-year-old boy who developed post-traumatic Dupuytren’s contracture, which was treated by segmental fasciectomy. The disease was histologically confirmed. We review the literature describing similar cases, and this case-report provides an important reminder for clinicians reviewing similar presentations that paediatric post-traumatic Dupuytren’s should be considered in the differential diagnosis and managed accordingly.

## Introduction

Dupuytren’s disease is a common disorder of the hand with a global prevalence of approximately 8% [1,2]. Despite extensive study, the exact cause remains unknown, although a number of environmental and genetic factors are thought to be involved [2]. It is also known that trauma, smoking, alcohol use, diabetes mellitus and epilepsy are associated with the development of Dupuytren’s [2,3]. Although it usually affects adults, it has been described in children, especially those with a strong family history of the condition [4]. Therefore, although rare, it should still be considered as a possible cause for the gradual onset of a flexion deformity in children.

## Case presentation

**Figure 1.**
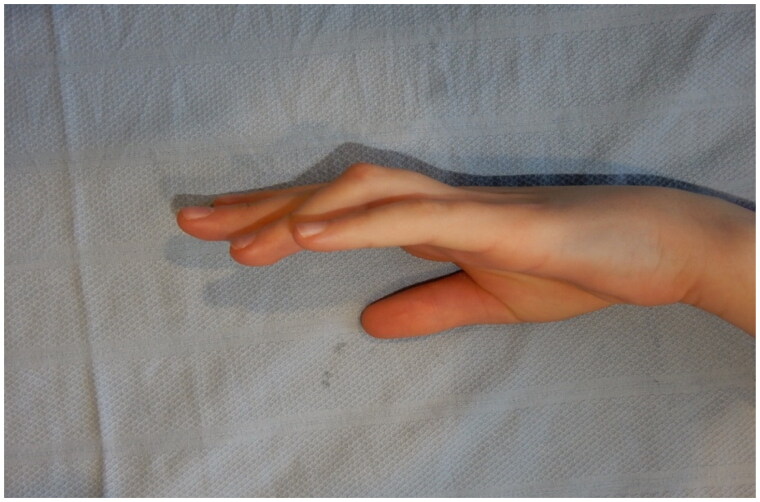
Lateral view of 45-degree flexion deformity of the left ring finger.

**Figure 2.**
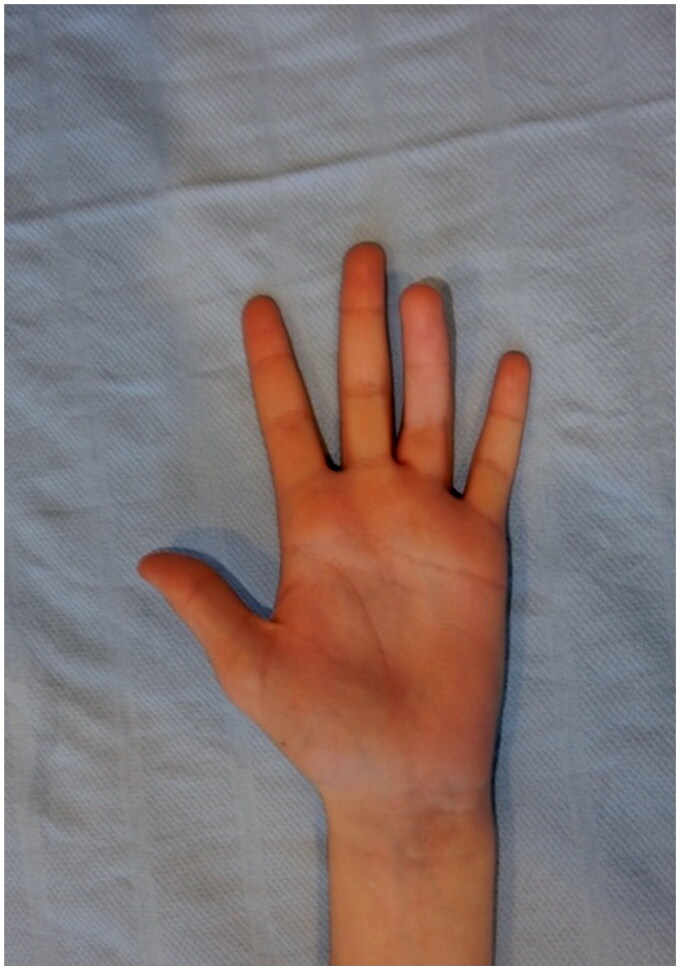
Anterior-posterior view of 45-degree flexion deformity of the left ring finger.

In November 2023, a 14-year-old boy presented to the plastic surgery department with concerns about the posture of his left ring finger and an increasing flexion deformity at the proximal interphalangeal (PIP) joint. He gave a history of an injury to the digit while playing basketball 5 years before. The patient was initially seen by his general practitioner in 2018 for the injury. Four years later, he was diagnosed with a flexion deformity of the left ring finger and in March 2023 a referral was made to the Orthopaedic service. An MRI scan of the finger showed a well-defined, irregular, soft-tissue lesion along the radial border of the finger proximal to the PIP joint, elevating the radial aspect of the of the extensor hood and touching the radial aspect of the flexor tendon. A similar soft tissue mass was also seen between the proximal phalangeal head and the flexor tendon. The differential diagnosis made by the Orthopaedic surgeons included a pigmented villonodular synovitis (i.e. ‘giant cell tumour’). So, they elected to refer the patient to the Plastic surgeons for further evaluation. Clinical examination by the Plastic surgeon confirmed the presence of a spiral cord of Dupuytren’s tissue on the radial side of the left ring finger, causing a 45-degree flexion deformity at the PIP joint (Figures 1 and 2). The patient was able to flex their finger fully and sensation was intact. In addition, there was a palpable nodule on the Flexor Digitorum Superficialis (FDS) tendon at the A1-pulley suggesting that triggering of the tendon might have contributed to the flexion deformity. There was no family history of Dupuytren’s disease and no other predisposing factors apart from the history of direct trauma.

## Management and operative technique

**Figure 3.**
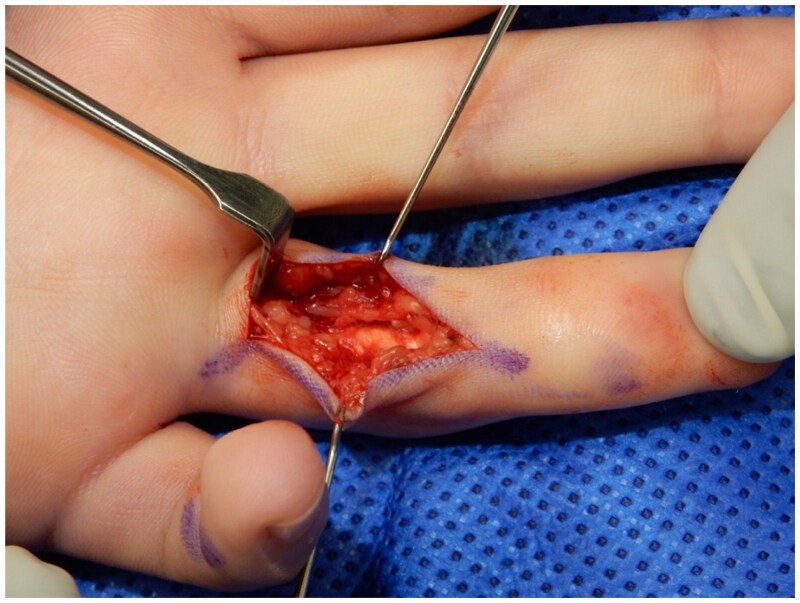
Spiral cord on the radial side of left ring finger.

Exploration of the left ring finger was performed under local anaesthesia (0.5% Marcaine with adrenaline − 10 mls as a ring-block). Through a Brunner’s incision, a spiral cord of Dupuytren’s tissue was seen, running along the radial aspect of the finger, next to the neurovascular bundle. The cord extended proximally to the MCP joint but not distally. A 1 cm segment of the spiral cord was excised (Figure 3), preserving the radial neurovascular bundle. A segmental fasciectomy was performed instead of a complete fasciectomy in this case as it achieves similar outcomes while being less invasive, having a quicker recovery and less pain following the procedure [5]. According to Ruettermann et al. (2021), the link between incomplete resection of the cord and recurrence of the disease compared to complete excision of the cord needs further investigation, and the previous studies have not provided evidence to explore this further [5]. After excision, the flexion deformity of the PIP joint was fully corrected and the skin was closed with vicryl rapide sutures. Histological examination of the excised tissue confirmed that this was a fibromatosis with no evidence of malignancy or atypia and consistent with Dupuytren’s disease.

## Follow-up

**Figure 4.**
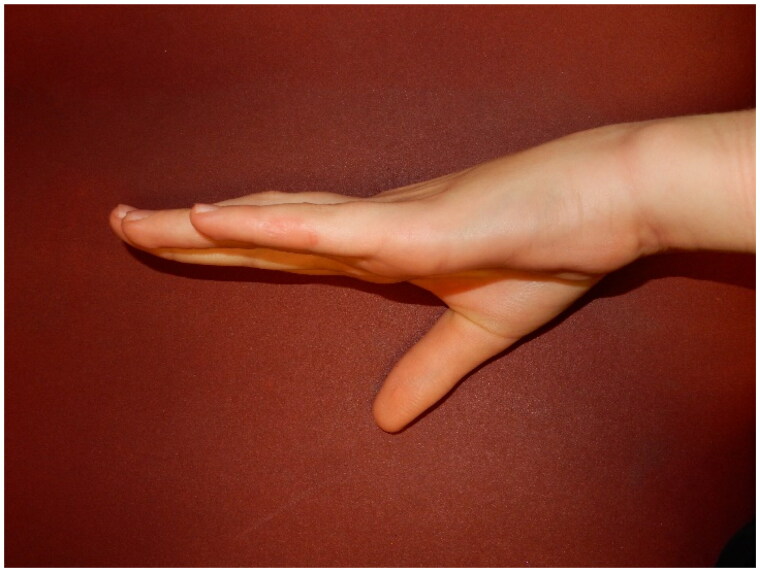
Lateral view of the left ring finger at seven-months post-procedure.

The patient was discharged on the day of surgery. A Zimmer splint (foam and aluminium) was applied to keep the finger in full extension at the inter-phalangeal and metacarpophalangeal joints. The Zimmer splint was replaced with a thermoplastic extension splint at 3 days post-op, and the patient was encouraged to begin mobilising the finger under Hand-Therapy supervision. The patient was also instructed to use the extension splint every night - indefinitely. According to Rodrigues et al. (2015), surgery is solely one aspect of treating the disease, and the recovery of full extension not only depends on the surgical approach but also on postoperative hand therapy, splinting, complying with rehabilitation and other elements such as the location of the cord and patient selection [6]. At 4 months and 11 days post-op, the therapists noted that he was complying with splinting, the incision had healed, and the active range of motion was 0°–90° at the MCP joint, 15°–90° at the PIP joint and 0°–70° at the DIP joint. At seven-months post-procedure, the patient was noted to be fully compliant with splinting, achieving full active range of flexion and extension (Figure 4), and was subsequently discharged. Ongoing daily hand exercises and night-time-only extension splintage have been advised.

## Discussion

Dupuytren’s disease is a fibroproliferative disorder that changes the palmar or digital fascial bands into cords, causing extension deficit and subsequent functional impairment of the hand [1,2]. This disorder has been known as the ‘disease of the north’ due to theories stating its origin in the Viking population and its common occurrence in the Northern European ethnicities; however, with time progression and the disease spread, it can affect patients from any background now [2]. There is no cure for Dupuytren’s disease; however, various interventions exist to improve hand function, and surgery is the intervention of choice in most cases [2]. Treatment options include Collagenase injections, needle and open fasciotomy, fasciectomy (segmental, limited and regional) and Dermofasciectomy, depending on the severity, location and extension of the disease [6]. Dupuytren’s disease pathophysiology resembles wound healing, and trauma can trigger an abnormal healing response, leading to the formation of fibrotic cords. Dupuytren himself considered trauma as a predisposing factor for the disease [2]. Elliot and Ragoowansi criteria was established in 2005 for recognition of Dupuytren’s contracture after acute injury following the identification of 333 cases of Dupuytren’s disease who had a previous acute injury as well as their own study of 52 patients who had a history of trauma before the disease development [7]. In 2005, Rayan and Moore reported a number of cases with ‘non-Dupuytren’s palmar fascial disease’, where they proposed that the leading risk factor for this condition is previous trauma or surgery as this was the case in 28 of 39 patients in their study [8]. The patient in our case study would not be a ‘‘non-Dupuytren’s palmar fascial disease’’ case as his condition was progressive, only affecting the digit without palmar involvement [8], Furthermore, histology subsequently confirmed Dupuytren’s disease in this instance. Our literature search revealed a total of 25 cases of paediatric Dupuytren’s disease in the literature [4,9–11]. This compares with estimated incidences of 12%, 21%, and 29% at ages 55, 65, and 75 years, respectively [12]. Only 2 of the paediatric cases we reviewed had a clear traumatic trigger [10,11]. Lee et al. (2023) reported a 14-year-old boy who developed a 40° extension deformity of the right ring-finger at the MCP joint which was treated with fasciectomy, full-thickness skin graft and post-operative Triamcinolone injections [10]. Similarly, Kibadi Kapay et al. (2022) reported a case of a 10-year-old male who developed a firm nodule in his left palm which was treated by fasciectomy [11]. Although clearly rare, post-traumatic Dupuytren’s does occur, and clinicians should consider this diagnosis as a cause for the gradual onset of a flexion deformity in children, after trauma.
